# Pneumocystis jirovecii pneumonia: a case report

**DOI:** 10.1186/s13256-024-04350-4

**Published:** 2024-02-12

**Authors:** Jacqueline Gri, Varun Jain

**Affiliations:** 1 Internal Medicine Program, University of Connecticut, Farmington, CT USA; 2 Department of Internal Medicine, Saint Francis Hospital, Hartford, CT USA

**Keywords:** Pneumocystis, Pneumonia, HIV, Immunocompromised, Infection

## Abstract

**Introduction and importance:**

Pneumocystis jirovecii (PJP) pneumonia is a serious life-threatening condition in immunocompromised individuals and is often associated with human immunodeficiency virus (HIV) + patients. We describe a case of PJP pneumonia which provided a diagnostic challenge in a patient who presented with no known risk factors leading to a delay in initiation of appropriate antibiotic therapy.

**Case presentation:**

A 71-year-old previously healthy white/Caucasian male presented with subacute hypoxic respiratory failure due to multifocal pneumonia with diffuse bilateral ground glass opacities with consolidations despite prior treatment with antibiotics and steroids. He was admitted and started on intravenous broad-spectrum antibiotics but continued to deteriorate, eventually requiring intubation and transfer to the ICU. Bronchoscopy revealed PJP and treatment was initiated, but the patient developed refractory shock and multiorgan failure, and ultimately died. It was later discovered that he was HIV-1 positive.

**Clinical discussion:**

PJP, as a potential cause of his presentation, was not considered given that our patient lacked any overt risk factors for PJP pneumonia. He continued to worsen despite broad spectrum antibiotic therapy and hence bronchoscopy was pursued. His clinical profile, in hindsight, was suspicious for PJP pneumonia and early PJP-directed antibiotic therapy may have prevented a fatal outcome, as in this case. There was an element of cognitive bias across multiple providers which may have contributed to the delay in treatment despite his rapid clinical decline while on conventional pneumonia treatment protocol. His diagnosis was later evident when his BAL-DFA grew PJP in addition to his low levels of CD4 and CD8 cells. He was found to be HIV-1 positive five days after his death; there was a delay in this diagnosis since all positive HIV tests from the hospital are reported as ‘pending’ until the presumptive positive sample goes to the Connecticut Department of Public Health State laboratory for the confirmatory test. PJP-targeted therapies were initiated later in our patient’s hospital course when the infection had progressed to refractory septic shock with multiorgan failure and eventual death.

**Conclusion:**

PJP pneumonia is a fatal disease if not recognized early in the course of illness, and the patient usually undergoes multiple antibiotic regimens before they are diagnosed and receive appropriate clinical care. The gold standard of diagnostic testing for PJP is by obtaining bronchial washings through a flexible bronchoscopy and the turnaround time for such results may take a few days to result. A significant proportion of patients may not have any overt risk factors of immunosuppression and early empiric treatment for PJP may be clinically appropriate as the delay in diagnosis may be associated with significant morbidity and mortality risk.

## Introduction

Pneumocystis jirovecii pneumonia (PJP) is a well-known condition often seen in immunocompromised patients, specifically associated with HIV positive individuals. It is classified as an obligate intracellular fungus; it develops from trophozoites that specifically attach to type I alveolar cells in the lungs and grow into cysts that contain sporozoites [1]. An adequate innate and adaptive immune system is necessary for clearance of the infection and prevention of alveolar damage. Alveolar macrophages are the resident macrophages that mediate the initial host response, the recruitment and activation of the inflammatory cascade and the cell mediated immune response. CD4 + T-helper cells activate monocytes and macrophages, triggering the humoral response and secretion of IgG for the opsonization and degradation of the organism. The inflammatory cascade leads to lung injury through metalloproteinases and free radical production. In patients with HIV, the CD4 + T-helper cells are depleted, and the immune response becomes dysregulated leading to severe lung injury and acute respiratory distress syndrome.

PJP incidence has dramatically decreased after the advent of antiretroviral therapy (ART) but is still most associated with HIV, especially low absolute CD4 count ≤ 200. Patients with HIV-negative PJP are usually those with hematologic malignancies, solid organ transplant recipients or those people receiving chronic glucocorticoids, chemotherapeutic agents, and other immunosuppressive therapies [2].

## Case presentation

A 71-year-old presumably immunocompetent white/Caucasian male, with a past medical history of hypertension and hyperlipidemia who was transferred from an outside hospital with worsening hypoxic respiratory failure requiring bronchoscopy. He was initially admitted to the outside hospital for progressively worsening respiratory failure over the past three days with increasing oxygen requirements. He had presented to the ED one week prior to the index hospitalization for similar symptoms and had completed an oral course levofloxacin and prednisone and sent back home. Unfortunately, his clinical condition worsened prompting readmission back to the hospital. He did not report any chronic respiratory problems however he disclosed having worked in the chemical industry for most of his life with exposure to “chlorine and other chemicals.” He was running three to four miles twice a week prior to the start of his symptoms and had run a half marathon three months prior to the admission. He reported three episodes of pneumonia as a young adult and, at the time, required excision of residual scar tissue for which no further details were available. Patient admitted to a ten pack-year smoking history in his twenties but denied alcohol or drug use. He had COVID-19 in 2021 but with no residual respiratory symptoms.

**Fig. 1:**
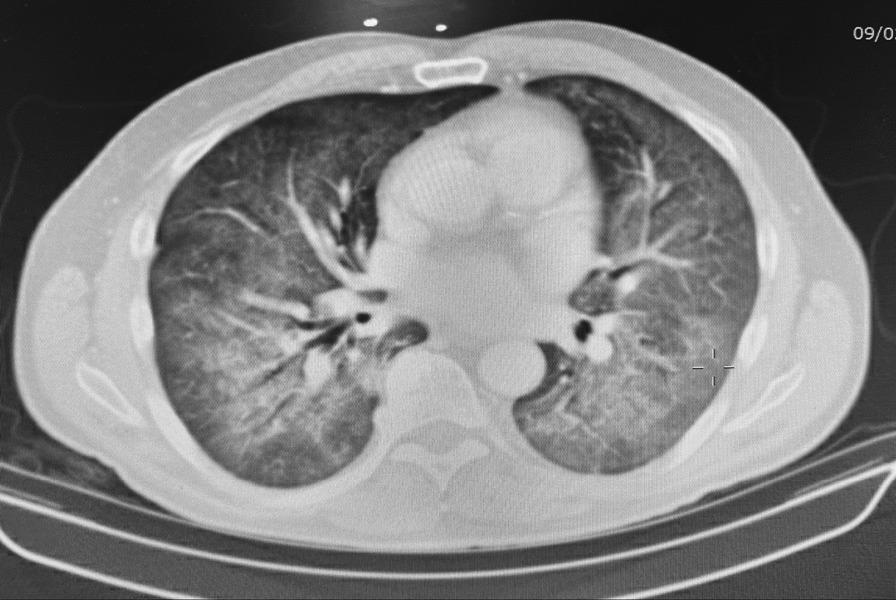
High resolution CT Chest with intravenous contrast. Notable for bilateral, patchy, and diffuse ground glass opacities in a batwing configuration

On presentation at the outside hospital, oxygen saturation was 85% on room air, which improved to > 90% with 4 L nasal cannula; he was otherwise hemodynamically stable, and his mental status was intact. Initial labs showed BUN/Creatinine 28/0.9, Na 133, bicarbonate 21, aspartate transferase (AST) 48 and alanine transferase (ALT) 109, lactate dehydrogenase (LDH) 662 and lactic acid was elevated at 2.3, brain natriuretic peptide was 118. White blood cell count was 16,000 and chest X-ray showed diffuse bilateral pulmonary consolidations. CTA of the chest was negative for pulmonary embolism but notable for bilateral ground glass opacities suggestive of atypical pneumonia, without edema or effusion (see Fig. 1). Blood cultures were obtained which remained negative. He was started on vancomycin, cefepime, levofloxacin, metronidazole and methylprednisolone which did not improve his symptoms and he was transferred to our tertiary care hospital for bronchoscopy after three days of quadruple antibiotic therapy.

On transfer to our hospital, he was noted to be significantly hypoxic and quickly escalated to high flow nasal cannula 100% FiO2, 40 L per minute. Given his concerns for atypical pneumonia, non-responsive to broad spectrum antibiotics, further immunodeficiency and rheumatological workup was ordered, including HIV panel, fungitell (1,3 beta d-glucan); these labs were not available until later during hospitalization. His Babesia, Legionella, Chlamydia pneumoniae, and Lyme serology were negative. His antibiotics were switched to intravenous vancomycin and cefepime and steroids were continued. Within 24 hour of his transfer, oxygen requirements increased to maximal high flow nasal cannula and he was urgently transferred to the medical intensive care unit on for elective intubation and flexible bronchoscopy. The bronchoscopy revealed no mucus or inflammation in the mainstem bronchi or lung fields. There was 40 cc of blood-tinged fluid after a lavage of 60 cc, with 7709 RBCs and neutrophilic predominance; cultures of lavage fluids were sent for analysis. Given these preliminary results, the infectious disease team initiated meropenem and vancomycin on day four due to continued proression of his respiratory failure. On day seven, his DFA of BAL fluid sample returned positive for PJP prompting the addition of IV trimethoprim/sulfamethoxazole. At this time, his further laboratory results became available including a fungitell titer > 500 pg/ml, decreased alpha and gamma immunoglobulin levels. Rheumatological work up including ANA, ENA, anti-CCP, RA Factor, myositis and anti Jo-1 antibody panel were negative.

Our patient’s ICU course was complicated with worsening renal failure due to acute tubular necrosis, development of acute respiratory distress syndrome and refractory septic shock. Despite initiation of appropriate antibiotic therapy his clinical condition worsened to develop end stage multiorgan failure. After multiple discussions with his family, he was made comfort measures and was died in the hospital.

## Discussion

Our patient’s presentation was very unusual as he was previously healthy with no reported personal or family history of immunodeficiency or chronic respiratory conditions. There was also no history of steroid use, malignancy, or immunotherapy. PJP diagnosis was established after the bronchoscopy fluid sample became available on day seven of hospitalization and HIV test was positive five days following his death, with a CD4 count of 3. HIV result was significantly delayed, and testing was not obtained earlier at the outside hospital due to presumed lack of risk factors. After transfer to our hospital, results were not available until weeks later as positive HIV results are sent to the Department of Public Health for confirmation before they become available. Unfortunately, the clinicians were not informed of a presumptive positive test and eariler treatment was not initiated. The USPTF has a grade A recommendation for one time HIV screening in adults, and more frequently for those at higher risk; it was unclear whether outpatient screening was ever done. Regular outpatient follow-up with a trusted PCP may have been lifesaving for earlier detection of HIV and initiation of antiretroviral therapy. Unfortunately, this case is one of many missed diagnoses of PJP pneumonia. (Miller 2018) conducted a retrospective study of 7656 cases of symptomatic PJP with and without HIV to determine the rates of diagnostic delay. It was found that 77.4% of patients without known HIV had a significant diagnostic delay relative to 18.5% in HIV patients. In addition, delayed diagnosis was also more likely to occur in outpatient settings. These findings suggests that patients’ symptoms often progress to the point of hospitalization before a correct diagnosis is made and proper treatment is initiated. Frequent outpatient follow-up, screening and accurate history through long-term doctor-patient rapport may be the next step to improve this delay.

Without a known history of HIV, a deeper exploration of the patient’s family history for congenital immunodeficiencies may be useful in determining other potential causes of an immunocompromised state. Nezelof’s syndrome or Common Variable Immunodeficiency should be considered [4]. Our patient also reported a history of working in the chemical manufacturing industry for many years. Fenga *et al.* (2017) discuss the effect of lead toxicity on decreased Th1 cell function; the mechanism is not certain although this may be due to impaired INF-Y biosynthesis. Serdar *et al.* (2014) conducted a cross-sectional study from NHANES 2003–2004 data looking at the association between polychlorinated biphenyls (PCBs) and organochlorine pesticides (OCPs) and effects on various cell lineages. It was found that higher blood levels of PCBs and OCPs were associated with lower leukocyte counts relative to other cell lineages. This exposure to various toxic chemicals for several years may have contributed to the extremely low CD4 count and disease severity.

The definitive diagnosis of PJP is made either through direct visualization of cysts or trophozoites via colorimetric/fluorescent stains, or with PCR. Diagnosis may be challenging without a bronchoscopy and biopsy, as sputum results may be falsely negative. Serum levels of 1-3-β-d-Glucan (through serum fungitell), a component of fungal cell walls, is often the initial test used to for a diagnosis when bronchoscopy is not feasible; however, it has limited utility as it cannot differentiate between different fungal organisms. Serum fungitell has a sensitivity of 80% and specificity of 63%, making it a decent initial test to direct further testing. When there is adequate suspicion for PJP, detection of serum 1-3-β-d-Glucan may be useful for assurance in starting antibiotic therapy empirically [7]. Prior to attempting bronchoscopy, imaging through chest radiograph and subsequently high-resolution CT scan is obtained. Characteristic imaging patterns are useful for hinting at the diagnosis and provide prognostic value as the degree of lung damage can be visualized and trended overtime [8]. Fig. 1 demonstrates the pathognomonic ground glass opacities representing interstitial lung damage secondary to interstitial inflammation and eventual formation of fibrous tissue and cellular debris.

Our patient’s diagnosis was made by bronchoscopy and BAL lavage, with results available on day seven; on that same day, the serum fungitell was also positive. BAL with fluid sample for PJP has a sensitivity of 97% and specificity of 85%; it is the gold standard for confirmation of diagnosis. Once PJP was detected, IV trimethoprim/sulfamethoxazole was added to the antibiotic regimen. Unfortunately, the patient already developed refractory shock by the time of initiation of appropriate antibiotic therapy. Given that our patient was initially placed on four different antibiotics at the outside hospital without clinical improvement, a modification of antibiotics therapy sooner would have been a reasonable approach, especially considering the characteristic imaging findings. It is unclear why the patient was started on anaerobic coverage with metronidazole, especially since there was no notable abscess or empyema on the CT chest. Our patient was also initially treated with steroids due to concern for undiagnosed interstitial lung disease (ILD). Given that there was no improvement despite the use of high dose steroids favored against the diagnosis of ILD and subtypes including cryptogenic organizing pneumonia (COP) and idiopathic eosinophilic pneumonia (IEP). [9]

Trimethoprim-sulfamethoxazole is the first-line medication approved for the treatment and prophylaxis of Pneumocystis jirovecii pneumonia. Benfield *et al.* (2008) conducted a systematic review of 29 studies evaluating the use of primaquine/clindamycin as a second line agent in the treatment of PJP. It was found that response rates were non-inferior to that of trimethoprim/sulfamethoxazole; however, IV pentamidine was significantly less efficacious. Based on the finding of this systematic review, initiation of either primaquine/clindamycin or trimethoprim/sulfamethoxazole earlier during therapy could have prevented worsening outcomes.

There are several cognitive biases that may have contributed to the delay in diagnosis of our patient’s PJP pneumonia. Confirmation bias is a possibility; the provider may have focused on confirming their initial diagnosis of community-acquired pneumonia and may have downplayed evidence that did not support this initial hypothesis. Anchoring bias is another strong possibility, where the healthcare provider may have relied too heavily on the patient’s lack of overt risk factors for PJP pneumonia and may have discounted the possibility of this diagnosis despite the presence of clinical symptoms and radiological findings. Finally, availability bias may have been an issue if the provider relied on prior experience with similar cases and may not have considered the possibility of a rare diagnosis like PJP pneumonia, particularly in an immunocompetent patient without overt risk factors. These cognitive biases highlight the importance of being aware of potential biases in clinical decision-making and actively seeking out alternative explanations for patient symptoms, particularly in cases where the initial diagnosis is not responding to treatment as expected.

## Conclusion

It is important to consider rarer or other unexpected diagnoses in patients who do not show clinical improvement on standard therapy. In this case, a presumably immunocompetent individual was admitted for subacute hypoxic respiratory failure and continued to clinically worsen despite adequate coverage; initially for community acquired pneumonia and later, hospital associated pneumonia, with imaging and laboratory findings being concerning for an atypical pneumonia including Pneumocystis jirovecii. A complete workup for atypical pneumonia was sent, however empiric PJP pneumonia therapy was not chosen due to lack of risk factors. By the time the results were available and targeted treatment could be pursued the patient had developed life threatening complications and died in the hospital. Early recognition and awareness of PJP pneumonia is essential and choosing to initiate empiric therapy in select cases would not be an unreasonable approach until the test results objectively confirm or reject the diagnosis which can otherwise lead to death or unfavorable outcomes. Early recognition should be especially considered in patients with chronic disease as the presence of infection and chronic disease can worsen the prognosis of this serious condition. [11, 12]

## Data Availability

Data available upon request.
